# The Significance of Emotions and Professional Relations for Accommodating a Web-Based Ulcer Record and Improving Home-Based Care

**DOI:** 10.3390/healthcare3010020

**Published:** 2015-01-22

**Authors:** Anne G. Ekeland

**Affiliations:** 1 Norwegian Centre for Integrated Care and Telemedicine, University Hospital of Tromsø, P.O. Box 35, N-9038 Tromsø, Norway; E-Mail: anne.granstrom.ekeland@telemed.no; Tel.: +47-952-66791; 2 Telemedicine and e-health Research Group, Faculty of Health Sciences, UiT The Arctic University of Tromsø, N-9038Tromsø, Norway

**Keywords:** web-based ulcer record, emotional agency, professional relations, innovation, improvement of care

## Abstract

Evidence of technological performance, medical improvements and economic effectiveness is generally considered sufficient for judging advances in healthcare. In this paper, I aim to add knowledge about the ways human emotions and professional relations play roles in the processes of accommodating new technologies for quality improvements. A newly-implemented, web-based ulcer record service for patients with chronic skin ulcers constitutes the case. After one year, only a few home care nurses were using the service, interacting with a specialist team. The result was disappointing, but the few users were enthusiastic. An explorative, qualitative study was initiated to understand the users, the processes that accounted for use and how improvements were enacted. In the paper, I expose the emotional aspects of the record accommodation by analyzing the ways emotions were translated in the process and how they influenced the improvements. I contend that use came about through a heterogeneous assemblage of ethical engagement and compassionate emotions stemming from frustration, combined with technological affordances and relations between different professionals. Certain aspects of the improvements are exposed. These are discussed as: (1) reconciliations between the medical facts and rational judgments, on one side, and the emotional and subjective values for judging quality, on the other; and (2) mediation between standardized and personalized care. The healing of ulcers was combined with a sense of purpose and wellbeing to validate improvements. Emotions were strongly involved, and the power of evaluative emotions and professional relations should be further explored to add to the understanding of innovation processes and to validate quality improvements.

## 1. Introduction

Chronic skin ulcers are characterized by delayed healing, cellular senescence and recurrent infections. They have been described as developing in conjunction with other health problems, such as obesity and diabetes, and are thus an increasingly common threat to public health and the economy in the U.S. and Western countries on a macro level [1,2]. An ageing population also accounts for the incidence of wounds. The economic, social and medical challenges call for a higher level of attention to understanding not only mechanisms underlying wounds, but also how advances in treatment come about and are validated. Chronic ulcer care is considered both prevalent and costly in home care. In 2002, Kobza and Sheuric found that inconsistencies in wound assessment and documentation and low usage of advanced products contributed to costs by resulting in lengthened healing time, more frequent visits by practitioners and low healing rates. As a potential remedy in this challenging situation, they analyzed a telemedicine service involving a two-way video visit, where a specialist assessed the patient and made recommendations for treatment. Results revealed improved healing rates and decreased healing time, number of home care visits and number of hospitalizations due to complications. Telemedicine was deemed viable for delivering quality, cost-effective care to ulcer patients in the home care setting [3]. In 2006, the acceptance of teledermatology was considered high in patients, home care nurses and ulcer experts [4].

Such considerations of cost effectiveness, evidence-based quality and the viability of telemedicine technologies in ulcer care set the stage for the development of a web-based ulcer record at the University Hospital in Norway (UHN). The ulcer team at the Department of Dermatology (DoD) were experiencing challenges, as described by Kobza and Sheuric above, and were looking for ways to improve and make services more effective [5]. After a three-year process, a pioneer service was implemented. By the latter part of this period, adaptation to Norwegian legislation and professionals’ needs had taken place [6]. In general, Norwegian legislation does not allow the use of mobile units with electronic patient records for privacy and security reasons. This ulcer record was not integrated with the general electronic patient record system of the UHN and contained only limited information about the patients involved; thus, there was a lower security level.

The web-based ulcer record had been developed in Denmark (www.pleie.net) and was intended to improve the interactions among dermatology specialists, general practitioners, home care nurses and patients and to make services more coordinated and consistent. The record operated via mobile units or computers and comprised databases, an ulcer analysis tool and an application for image exchanges and free text sharing between participants. The service included a specialist ulcer team, including a dermatologist and trained ulcer nurses, who would advise home care nurses, general practitioners or patients through the record. The specialists at the UHN initiated the establishment of the record, and it was operational from January, 2011.

The team was optimistic and introduced the new service to all home care nurses who visited with their patients for specialist services. After one year, however, only four home care nurses at two different sites had used the record with one patient each. The ulcer team was surprised by this disappointing result, since evidence for quality, technological usefulness and effectiveness had been demonstrated in earlier studies. They also expressed that the few users were very enthusiastic about the record.

This experience was the background for an explorative, process-oriented, qualitative research project initiated in 2011. This paper is based on one branch of the project, where the purpose was to, from the few users’ points of view: (1) assess and understand professional users; (2) likewise, the processes that accounted for use at the few sites; and (3) likewise, the improvements that were obtained. A different branch of the project addressed the experiences of patients who took care of their own ulcers, and a paper has been published [7].

The purpose for the branch reported here was not to understand why uptake was low. Rather, in order to direct possible cumulative development, the goal was to understand how use came about and what it did for the few users and quality of services. The practical purpose was to learn from users and to help improve services as they developed. This formative research approach was designed to produce systematic knowledge and to inform potential users of the experiences from actual users [8]. Enthusiasm among the few users was discussed by the specialist team as crucial for the future viability and success of this service innovation. Such reflections point to emotional issues as an ingredient in success, adding to evidence-based medical rigor, technological usefulness and effectiveness. Such emotional aspects of accommodation processes and quality advances are understudied. Understanding the inter-relations between emotions, the interaction between professionals, the use of new technologies and medical improvements therefore became a focus.

To fulfil the potential benefits of new technologies, the transparency of processes and the results through ongoing evaluation and feedback of their continuing improvement are important [9]. Addressing emotional engagement and social relations contribute to transparency and add new ingredients for understanding use. Therefore, the academic purposes were to understand how emotions were involved in the alignment of processes accounting for use and how this involvement helped enact specific improvements. This purpose also implied an analysis of the relations between processes and outcome.

The research question was:

How were emotions and professional social relations involved in the accommodation of the web-based ulcer record and in the advances of ulcer services?

I argue that emotions and relations between professionals are important influences for making the most of innovations directed at improving services. Therefore, the roles of human emotions and professional social processes for establishing grounded advances in healthcare should be given more attention. The paper exposes emotions conveyed in the empirical process of accommodation of the record and analyses improvements and interconnections referring to theoretical positions.

## 2. Methodology, Methods, Material and Introduction to Analytical Perspectives

The purpose of framing the research question to illuminate the process and results is to improve the understanding of the mix of influences that produce the specific results of innovations with advantages beyond the current practice [10]. Unpredictable contexts and confounders may lie at the very heart of diffusion, dissemination and implementation. Emotions and relations among professionals and between professionals and patients are unpredictable, but addressing multiple elements and interactions that arise in particular contexts and settings can improve the understanding of what determines the success of a dissemination initiative.

A systematic review of shared decision-making in clinical practice revealed that there was very little known about health professionals other than physicians on this subject [11]. Use of the web-based ulcer record is also about shared decisions. By investigating the interaction between the record, the home care nurses and the specialist team at UHN with an emphasis on the experiences of the home care nurses, their perspectives will also be added to the study of barriers and facilitators of new information and communication technologies (ICTs) in healthcare.

Within the socio-material network approach that underlies the study, all entities involved, including technologies, are considered in a state of mutual, simultaneous influencing. As such, the approach leans towards actor-network theory [12,13]. The majority of existing research and literature on the utilization of electronic patient records in healthcare within this theoretical universe pays attention to how heterogeneous, human and non-human elements relate and make up transformations in empirical settings [12,13,14]. The social construction of the technology perspective argues that people actively shape technologies by the meanings they give to them [15]. However, no specific attention has been paid to human emotions and values, social relations and the ways they are intertwined with scientific rigor and materiality and the ways they may influence both the processes of utilization and the results [16]. This paper aims to add to the understanding from this perspective.

The recruitment strategy for the research project was worked out in collaboration with the specialist ulcer team. The team was consulted in the development of the project protocol and agreed to collaborate. The additional selection of individuals for examination took place when professionals from home care or general practice visited with their patients. Nine professionals and six patients were included in the study and interviewed. The six interviewed patients took care of their own ulcers at home. We did not interview the two patients who were treated by the home care nurses. These two patients were fragile and had cognitive impairments; therefore, we considered it unethical to approach them for interviews. This article is mainly based on the experiences of six professionals: four home care nurses in two home care districts and two specialists from the ulcer team at the DoD, UHN. Three other interviewed professionals represented a district healthcare center. Their experiences were not directly included, as they did not use the ulcer record for patient care. However, these three expressed enthusiasm as a core characteristic; thus, their points of view were considered in the decision to focus on the role of emotions.

The interviews with the four home care nurses took place after each had used the record for ulcer treatment of one patient in each home care district over periods ranging from four to six months.

Frequent meetings were held with the specialist ulcer team during 2011 and the first six months of 2012. These discussions served as occasions for new data on the process of implementation and preliminary interpretations of the material collected. The specialists on the team were interviewed formally on two occasions: once at the beginning of the research project in February, 2011, and once in December, 2011.

The professionals were first asked about the treatment and care procedures before the introduction of the record, their attitudes towards the procedures, as well as their expectations of the record. They were then asked in detail how they used the record and how they experienced differences from previously established practices. The professionals were also asked to indicate the most important improvements in care as a result of using the record. The interviews were qualitative and open-ended. They were semi-structured in that the participants were first asked to give free accounts of their experiences and to later be more specific regarding technological affordances; meaning, the qualities of the record that allowed the users to perform new actions and that facilitated cooperation, the division of labor and roles, care procedures, knowledge and improvements. The interviews were digitally recorded and transcribed. The number of professionals interviewed was low, but they constituted the total of users within the period.

A task force comprising a project leader and two representatives from a security team concerned with legislation and ethics collaborated with the Danish company and the specialized team to implement and maintain the record. The researchers had access to meetings and discussions with this group on several occasions before, during and after the research period. These discussions were also important for continued updates concerning the record and how it was introduced, improved, accommodated and utilized.

Subject areas and perspectives for analyzing the process, outcomes and the relationships between them were gradually developed along with the interviews and frequent discussions with the ulcer team and task force. The subject areas and perspectives are briefly mentioned below. A development from frustration to enthusiasm following the use and interaction between professionals pointed to a dynamic movement towards a normative “future good” situation, collective action and emotional engagement. The analysis of the process of accommodation is therefore inspired by concepts from the theory of social movements and uses these concepts, which were developed by Tamotsu Shibutani [17].

The second discussion section addresses quality improvements. Two inter-related discussion themes were developed. The first theme refers to the advice given to home care nurses and the tensions between emotion and reason and value and fact in the judgment of actions. In his recent book, Andrew Sayer discusses the importance of acknowledging a person’s relation to the world as something of concern [18]. He contends that people’s subjective feelings are not just “free floating values” or expressions projected onto the world, but they are feelings about various events and circumstances and are not merely subjective. They reflect that people are social beings, dependent on others and necessarily involved in social practices. Martha Nussbaum’s theories of the intelligence of emotions and emotions as judgments of value and importance also inspired the analyses [19,20]. The second theme concerns standardized *versus* personalized care as conceptualized by Timmermans and Berg [21,22]. In the case discussed in this paper, the affordances of the web-based ulcer record included standards for representing images and displaying historical events, as well as standards for communication. The section is wrapped up by a discussion of emotions and social relations as supplements to the evidence of technological performance, medical improvements and economic effectiveness for understanding and judging innovations.

## 3. Results

Empirical findings and users’ reflections, actions and behaviors are presented according to the research question: How were emotions and professional social relations involved in the accommodation of the web-based ulcer record and in the advances of ulcer services? Results are presented in three subsections. These include accounts of: (1) the nurses’ descriptions and evaluations of the situation before the record was introduced; (2) the process of using the web-based ulcer record; and (3) the perceived and enacted improvements.

### 3.1. Emotions and Values: Vacillation and Frustration

A nurse’s emotional state is closely related to his or her patient’s emotional state. Therefore, following is a brief introduction of the patients in the study. One of the patients was a man in his late sixties located in District 1. He had had chronic wounds for many years, as well as several other conditions. He was described as a typical ulcer patient by one of the home care nurses, who indicated that his treatment was difficult: “It has been so bad that they wanted to take his foot”.

The other patient was a 60-year-old woman in District 2 who had two ulcers for a total of more than four years. The first one occurred in 2004 and healed. A new ulcer occurred in 2009. It healed during the summer of 2011 after use of the record for four months. The nurses informed us that knowledge about diagnoses had been difficult to obtain: “It was never known what kind of ulcer it was. They (the specialists) defined it as an ulcer with a possible circulatory problem with venous backflow”.

One nurse commented regarding advice from GPs:

They have the most knowledge about heart failure, diabetes, stroke and general conditions. I wanted her to use support stockings. She did not even look at them since the doctor had told her she did not need them. And you know, one patient had an ulcer for one year, and they did not even inform us. We even experienced amputation of toes before we were informed.

The nurses described feelings of perplexity and irresolution connected to the various advice from GPs. The nurse who commented about the GPs also explained that differing advice from various professionals made the nurses feel insecure within their districts:

We tried different methods, strong steroids, silver; we tried everything. We contacted the general practitioner, the local hospital and spoke with nurses who had experience with different bandages. We were puzzled and insecure. Different messages caused uncertainty, loss of initiative, reduced uniformity and continuity of treatment. The patient had been to the hospital several times. They had nothing else to give her than surgery. She did not want that. We were doubtful and needed someone to discuss it with. It was a very bad period. The ulcer grew worse, we lost faith and she lost faith.

The home care nurses reported challenges obtaining consistent knowledge of the kinds of ulcers they were treating and the best treatments to administer. They also expressed frustration with the diverging advice from specialists adhering to different guidelines and enacting different knowledge practices, skills and preferences.

Within District 2, nurses also reflected on their internal practices. They were discontented with the ways they kept track of interventions and evaluated their effects. They communicated fear:

It is a difficult job to remove bandages; we are not sure of how much we can touch the wound, what can be removed. We are afraid that we can cause damage and pain. There are many discussions; zinc or no zinc around the scabs, what kinds of bandages, expensive or not expensive? And you know, in our district, altogether 10 different nurses have taken care of ulcers, and they do things slightly differently. It is so difficult to know what works and what does not work when we do not know exactly what has been done over time.

Another nurse then commented:

Home care is not trained well enough for ulcer care; knowledge about what works is not consolidated. Who does what and who knows what works? Ulcer care is an area of low priority for training and low status. It also lacks organizational coordination. These aspects are connected.

An evaluation of the users’ discontent with their own practices, the advice they were given from collaborating partners and the knowledge base and status of their work revealed that the emotional states of the users were characterized by feelings of frustration, disappointment and vacillation.

### 3.2. The Web-Based Ulcer Record, Professional Relations and the Future Good

The home care nurses documented the ulcers once or twice per week and sent images and text once per week. They described any changes in each ulcer’s appearance and edges and then sent requests. They received responses immediately. The nurses were not familiar with reporting in the beginning and claimed that they were somewhat anxious. They reported feeling unable to find the right words, but that using the record to send images and using the tool to measure and describe the ulcers allowed the nurses to be more precise:

We really put in efforts to express ourselves and felt obliged to write good reports. It gets more serious when we have to report. We need to really consider what we write. It is easier and more definite when we can use both images and text. We become more observant concerning smell and how the ulcer looks.

The nurses also described situations where different professionals gradually strived to understand one another. The nurses explained their use of the record and the responses from specialists’ in terms of finding out what was expected of them and what to expect from the experts concerning future improvements. In District 2, one nurse explained:

We felt confident after a while. They use a different language than we do, which is more specialized, and we were insecure in the beginning, but managed to understand each other. They are good at explaining. They, too, put effort into explaining what the different things mean, the words they use, so we have learned a lot.

The nurses also reported knowledge and words were as important as images in their communication with specialists. According to a nurse in District 1, “The specialists are more vigorous than us. We learned to be more fearless, to scrape off the edges of the ulcer, to remove dry scabs and look beneath them”.

The nurses seemed to recognize their own anxiety, which they also communicated. Communicating anxiety sensitized the specialists. This way, they explored new opportunities for improving skills and were “on the move” in a purposeful direction. The nurses were enthusiastic about the ability to communicate meaningfully with the specialists. This was of great concern to them and a significant motivating factor in record use.

The standardized affordances of the ulcer record for storing and revisiting historical images, the measurement tool and the free text descriptions of interventions were valuable for clarification of what the nurses and specialists could expect of each other.

In the home care district where up to 10 different nurses had attended to ulcers, the staff managed to keep track of the most effective treatments and interventions: “We documented images, questions and reports once or twice a week and sent them once a week.” In their accounts, the nurses communicated a motivation to improve skills and crystallize purposive interventions; therefore, indicating that they were supported by the standardized affordances of the record and the situation in the district. The results of professional interventions were verified by empirical observations of the ulcers according to the developments documented in the record: “We used the opportunity to discuss the registered images and descriptions internally in the district. Were there changes over time? We read earlier answers together and consolidated our understanding”. The nurses’ accounts of the value of images and text combined, as well as the motivation to understand their professional collaboration partners were supported by the specialist team: “It is important to possess both knowledge and concepts in addition to images. Live images offer an opportunity for specialists and home care to confirm a common understanding and the development of common expressions”.

The future of ulcer care was discussed in terms of the need for collective action, more attention and higher status. One nurse stated:

We should have had the opportunity for more skilled people at all levels. The doctors should be more interested, and it should be a more developed specialty. The local communities should provide the technological equipment and be responsible for the training procedures. Teams responsible for ulcer treatment should be developed in each district, and they should be given education and resources.

The latter statement introduced the services’ relations to policy incentives, resource allocation and distribution.

The nurses’ visions were supported by the team of specialists:

The patients need collaboration between specialists and primary care, because knowledge and competence in each of these practices are not sufficient. It is necessary to make responsibilities and division of labor clear to improve communication and coordination between collaborating partners.

### 3.3. Improvement of Interventions, Medical Outcome and Self-Concepts

#### 3.3.1. Changing Regimes and Closing Gaps

After undergoing treatment at the UHN, a female patient was encouraged to believe that her ulcer would heal with time and effort. She was accompanied by a nurse who had been educated in the use of the ulcer record by the specialist team. The patient felt confident, and, on the nurse’s return, the other home care nurses in her district were encouraged and reassured to provide purposeful services:

We changed regimes in collaboration with the UHN when we did not see the effects of what we had done. When we came home, we gave her close attention and active care twice a day with the use of the ulcer record. We put the same nurses on the case, so the number of people attending to her was reduced. This concerted effort reversed the bad development we had seen in the ulcer.

#### 3.3.2. Detachment from the Current Order

One of the nurses expressed happiness about the development of the attitude of a male patient:

He used to go to the UHN for his treatments. He did not want to involve us, but to take care of his own ulcer. He had a tendency to keep information from us. Now, after he saw that we were determined, there is a new tendency, an open dialogue. No information slips away from us now. He sees that things are better. It is more positive now.

#### 3.3.3. Strength and Purpose: Self-Respect and Ulcer Healing

Practices, attitudes, knowledge and emotions were altered in the district where up to 10 people had attended to the patient. The nurses intervened faster and with a stronger sense of purpose:

We go in earlier, and it goes for our extras, too. They have been more observant about ulcers. Now, it is more serious and important work to them. It is because of the ulcer record. If we leave, the ulcers may grow. Having confidence means a lot, plus the attitude; that somebody says they have competence and resources. We really reached a new perspective on ulcer treatment; it is exciting. We have new knowledge; we know what to look for; we see improvement and dare to change procedures, because we know what works and not.

The nurses also talked about their feelings of confidence, wellbeing and self-respect. They related these feelings to being able to execute what they expected from themselves as professionals. One nurse said:

We have become more confident and reassured. We dare to remove scabs, to be more direct in our actions. It is expensive and intensive, but this really can prevent ulcers from growing bigger, avoid amputation and more expensive specialist interventions. There are no hocus-pocus interventions, but regular and concerted procedures. She (the patient) was confident that home care could do it. The pain that she had felt before when I touched the ulcer disappeared; she dared to let go of the fear of pain. She could see that somebody cared and was serious; the doctors and nurses had positive attitudes, and her attitude changed. The ulcer had grown for two years earlier. Now, it healed during a few months.

Roles and responsibilities changed in this process. The way these changes crystallized was also explained by a nurse:

I have become the responsible person within the group (of home care nurses). I prioritize ulcers, and it is easy for me to consult the specialists. I have become responsible for in-house training, and I feel obliged to take more responsibility.

The nurses demonstrated a reinterpretation of experiences, new behaviors and meaning, self-respect and strength. A sense of wellbeing emerged as opposed to the frustration they had expressed at the outset.

### 3.4. Summary of Results and Introduction of Discussion Themes

The results section first pointed to the users, their emotions and the process that was enacted. This section discusses the change process as originating in collective discontent [17]. By elaborating concepts of the theory of social movements, accommodation is further discussed as a translation process wherein professional interaction, and the affordances of the web-based ulcer record were included. I contend that the empirical process was an enactment of solidarity and responsibility.

At the outset, varying advice was associated with frustration. Use of the record system induced agreement and a sense of direction. The discussion of quality improvements first concerns advances that came about through mediation between the different knowledge practices. The current abundance of evidenced-based medical guidelines may facilitate the development of different practice cultures and put a growing pressure on interpretations, as reflected in the results. Such pressures may reinforce ethical and emotional aspects of judgments, closely connected with a discussion about advances caused by reconciliation between medical facts and rationality, on one side, and emotions and subjective values, on the other. The nurses experienced the delivery of varying medical advice as causing vacillation, and the emerging change towards concerted actions and healing ulcers was accompanied with a sense of purpose, thereby pointing to a close relationship between fact and value for judging improvements. 

Technological standards were strongly involved in both the advancement and personalization of care processes. The second discussion concerns quality improvements related to standardized* versus* personalized care. The two discussions are combined to point to the strong relationship between rational and emotional judgments. The healing of ulcers was combined with a sense of purpose and wellbeing that validated the improvements.

## 4. Discussion

### 4.1. The Processes of Accommodating Use: Dislocations and the Agency of Collective Emotions

Collective emotions of frustration, an urge for change and valuations of a partly utopian and normative “future good” have been described as driving forces for change [17].

Dislocation is a phenomenon that arises when different social units in a given community adapt differently to problematic situations. Working at cross purposes, as described by nurses, is an enactment of different adaptations to the problematic situation of diagnosing and treating ulcers. The situation where up to ten homecare nurses had performed incongruent procedures enacted social dislocation and incongruity within one of the districts. Social dislocations, emotional stress and uncertainty are at the core of change: “When dislocations happen, misunderstandings arise; people work at cross purposes, even when they are acting with the best of intentions. Accusations of fraud and indecency are made and incongruities and frustrations mount, many more dilemmas develop” [17].

Accusations of fraud were not directly communicated. Rather, home care nurses expressed frustration, low priority and insufficient knowledge on the part of GPs and the local hospital. They indirectly communicated that some professionals they expected to be responsible did not take their responsibility seriously. From this position, dilemmas were communicated.

Kierkegaard described fear as a driving force for moving from a frustrating here-and-now situation to a future better situation; fear strikes those who anticipate unmanageable demands [23]. The nurses’ accounts of disappointment from not being properly advised point to a feeling of not being able to manage the demands they put on themselves for ulcer care. The nurses failed to perform their work with excellence. Responses, such as disillusionment, helplessness and identity confusion, emerged. The feeling of inadequacy seemed to be reinforced by the specialists’ delivery of varying advice. This situation was a source of alarm, as nurses expressed not being able to concentrate, growing feelings of uncertainty and an increase in random or vacillating behavior.

The nurses’ use of the web-based record is an enactment of seeking support to alter their practices. A change in a formal norm can occur from an urge to find out what is expected and what to expect from others and is a mental process originating in the participant’s attempt to change his or her self-concept. The nurses and the specialized team made extensive efforts to understand each other and to document and communicate what they had done.

The nurses’ accounts of discontent and anxiety, their openness and motivation for change, their attempts to find out what was expected and what to expect from others, as well as both teams’ adjustment of their efforts characterize the emotional state and the social relations involved in the emerging change processes.

### 4.2. Enrolling the Record, Professionalism and Excellence in the Processes

The nurses used standardized tools for measuring and communicating ulcers and protocols for storing and retrieving historical images and text. These technological affordances were clearly vital for their skill development, common purposes and verifiable interventions. The technologies mattered for reconciling patchy and incomplete interventions.

A socio-material network of relations was enacted according to the nurses’ and specialists’ accounts of how different units possessed and practiced different procedures, skills and interventions and how their interactions through the web-based ulcer record gradually aligned these interventions. Their efforts and actions to continuously adjust their language and make interventions concerted and purposeful reflect what mattered to them, involving emotions and a dynamic trajectory towards their understanding of excellence [16,17]. Patterns once accepted disappeared, and old procedures were changed. A sense of purpose emerged, and the number of nurses attending the patient and collaborating with UHN was reduced. New ways of understanding and executing professional social transactions were produced.

### 4.3. Utopian Visions and Cumulative Impact?

The nurses’ visions of ulcer care as a specialty with higher status and the specialist team’s account of the need for integration and consensus represent a utopian vision conveyed by professionals in the larger ulcer community. Improvements were made with a gradual and modest cumulative impact. Expectations about oneself and one another were displaced. Alterations in customs involved a change in outlook. What the professionals took for granted as natural changed.

The agency of collective emotions and values and the dynamic trajectory evolving through efforts to fill the gap between a situation of frustration and vacillation and future excellence portray the processes of accommodating the ulcer record. The ulcer record, which provided affordances for the development of standardized skills, the interaction among the professionals and the emotional and value-laden agency were combined. Evaluative emotions stemming from frustration, ethical values, professional knowledge and future orientation were influences embedded and mediated within a socio-material network. Professionalism in skills, procedures and language was included, and this network changed and enabled purposeful interventions and processes, entailing empirical evidence of healing ulcers.

### 4.4. Improvements

Regarding the proposal of surgery and varying advice concerning interventions, the data provides examples where advice or interventions defined as factual emerged as partly subjective and emotional, whereas ethical evaluations turned out to be rational in that the healing of ulcers occurred. In the following sections, the alignments of fact and value, reason and emotion and standards and humanist care are discussed as products of, and producers of, practical reason.

### 4.5. Reconciling Tensions between Fact and Value and Reason and Emotions

Andrew Sayer discusses the importance for social science to acknowledge a person’s relation to the world as something of concern [18]. He contends that people’s subjective feelings are not just “free floating values” or expressions projected onto the world, but feelings about various events and circumstances that are not merely subjective. They reflect that people are social beings, dependent on others and necessarily involved in social practices. The challenges and quality improvements described in the case of the web-based ulcer record reflect relations between fact and value and reason and emotions. By failing to acknowledge people’s evaluative relation to the world, Sayer goes on to argue that social science might produce alienating accounts of social life.

The account of the ulcer that had grown for two years, but was healed within a few months of implementing the system reveals that the nurses’ evaluative relations, as well as their empathy and ethical considerations were closely related to factual observations. The account also demonstrated a connection between the facts of pain, the attitudes of the patient and nurses and the nurses’ actions. Pain seemed to be less painful, because the patient dared to let go of the fear of it, and inducing pain became less emotionally painful for the nurses when they believed in what they did.

The sense of wellbeing that both professionals and patients experienced as the movement proceeded shows the importance of emotions and everyday normative judgments as part of rationality: “Only by entering into the nature of the implications of our behavior for wellbeing can we fully explain the ethically evaluative character of human experience” [18].

Practical reason involves fact and value, mirroring one another. The empirical facts, the healing of ulcers, documented by technological affordances, images and descriptions of the ulcers that changed over time, combined with nurses’ confidence in approaching the patients and their feeling of wellbeing and strength as the ulcers healed are enactments of reconciliation between fact and value, reason and emotion. Practical reason was validated by the nature of the implications of the behavior for healing ulcers and wellbeing.

### 4.6. Closing Gaps between Standardized and Humanist Care

Standardized procedures were proposed by the local hospital. The proposal of surgery to the female patient was substantiated by an assessment of the ulcer. The patient rejected this proposal, and the home care nurse was sympathetic. The nurse clearly communicated doubt and evaluated her relation to the patient’s rejection of the proposal. She considered the proposal as connected with the repertoire of the local hospital and as neither an objective/evidence-based decision nor a personalized one. Accounts of varying advice from different professional actors caring for the same patient point to challenges concerning standards and rationality. In a common language, standards denote rationality, and humanist or personalized care denotes values and ethics. The ways the web-based ulcer record was used sensitized the reconciliation of possible contradictions between standardized and humanist care. Technological, organizational and procedural standards aligned with one another and then aligned with personalized and humanist care.

The nurses in the second district described a lack of coordination as a major problem connected to different professionals’ prescriptions, execution and control of various procedures based on formal responsibility, skills, interest and evidence-based guidelines. This was presented as one of the causes of frustration and vacillation. Hands-on knowledge, emotions of concern and relationships helped nurses crossbreed different advice. Combined with the options for expert consultations and the technological standards allowing for the examination of historical images and development over time, use of the record helped make services relevant, grounding them.

Personalized medicine and care involve evaluative relations and silent knowledge, and standards can be considered to carry the script of facts and reason [24,25]. The ulcer record facilitated the communication of reports and the structuring of text and images, thereby helping the nurses who strived to change expectations about themselves with respect to language and reporting. Their confidence and strength grew. Use of the record collected and enhanced standardized knowledge and skills and propelled them towards purpose and meaning.

The record provided for the standardization of text and image communication and storage, as well as a tool with which measurements and colors could be analyzed. These affordances helped the professionals perform more concerted, personalized and effective interventions. The accounts indicate that professional knowledge, skills, guidelines and standardized procedures were exchanged between collaborating partners and frequently tested against empirical documentation and emotional judgments. Professionalism was consolidated by connecting different standards with empirically-observed changes in conditions. This kind of professionalism was highly valued by nurses and the specialized team. The professional relations, fuelled by the demonstrated ethical engagement and compassion, helped the practitioners utilize standards in ways that reconciled possible tensions between standardized and humanist care.

### 4.7. Validating Advances

Demonstrated utility is considered key for validating quality. Utility was demonstrated through the communication that took place between units, resulting in the sharing of a larger, common purpose. The sharing of historical images and observations of healing ulcers were elements in the validation of the grounded character of the changes, as were the wellbeing of nurses and patients. Interventions were congruent and language concerted with the specialists and guidelines. The historical images supported the practical evidence of healing ulcers.

Accepting new roles and responsibilities and making claims to others enact new stages in development processes, which are manifested in social change and reconstruction [17]. Nurses grew in confidence. Confidence and purposeful interventions began to replace confusion and lack of direction. Skill improvement, concerted and purposeful actions and renewed self-respect emerged*.* Nussbaum argued that emotions play a crucial role in individual actors’ judgment of values [19,20]. I contend that the intertwinement of emotions with (1) standards and technologies allowing the assessment of the results of interventions through historical images, (2) medical facts in terms of healing ulcers and (3) relations with humans conveying different medical skills and knowledge aligned to validate practical reason.

## 5. Conclusions and Future Direction

Accounts of discontent in ulcer care and the processes mobilized for improvement were analyzed using a socio-material network approach. The ways the web-based ulcer record was utilized contributed to the co-development of change processes and demonstrated improvements in ulcer care and wellbeing of professionals.

The process connected and crossbred professional units, dualisms, standards and measurements. Two issues concerning quality improvements (within which challenges concerning quality have been strongly debated) were brought to the foreground: These were: (1) the tensions between emotions and values, on one side, and rationality and facts, on the other; and (2) standardized* versus* personalized care processes.

I argued that the change processes were enacted as the effects of dynamics between technological standards, rationality and facts, values, emotions and humanist care, through the technology-mediated interaction, which served to ground improvements by reconciling practices that had been working at cross purposes.

Practical reason was enacted, denoting improvements that responded adequately to the needs of patients. Frustration, vacillation and a sense of being unethical were translated into purpose, strength and wellbeing. By exposing the roles of emotions and social relations through the use of the web-based record, the importance of these two factors in change processes and for judging advances was clearly demonstrated.

Exploring emotions together with values and normativity and their roles in generating results can deepen the understanding of innovation processes. Including emotions and ethics as ingredients in assessments of processes and results is also a way of humanizing the accountability of each participant who works collectively in health services to make complex judgments about what is a good service. Focusing evidence, standards and effectiveness can be a way to disclaim the role of everyday human responsibility. Ulcers are a complex and increasingly common threat; therefore, drawing on the energy of evaluative emotions and professional social relations should be further explored as resources for improving services. Human emotions in interaction with new tools and rational considerations are important resources that should be further explored in innovation processes and for judging quality improvements.
